# Integrating network ecology with applied conservation: a synthesis and guide to implementation

**DOI:** 10.1093/aobpla/plv076

**Published:** 2015-07-10

**Authors:** Christopher N. Kaiser-Bunbury, Nico Blüthgen

**Affiliations:** Ecological Networks, Department of Biology, TU Darmstadt, Schnittspahnstr. 3, 64287 Darmstadt, Germany

**Keywords:** Adaptive management, biodiversity conservation, ecological integrity, ecosystem functions, indicators, interaction networks, islands, pollination

## Abstract

Ecological networks are a useful tool to study the complexity of biotic interactions at a community level. Advances in the understanding of network patterns encourage the application of a network approach in other disciplines than theoretical ecology, such as biodiversity conservation. So far, however, practical applications have been meagre. Here we present a framework for network analysis to be harnessed to advance conservation management by using plant–pollinator networks and islands as model systems. Conservation practitioners require indicators to monitor and assess management effectiveness and validate overall conservation goals. By distinguishing between two network attributes, the ‘diversity’ and ‘distribution’ of interactions, on three hierarchical levels (species, guild/group and network) we identify seven quantitative metrics to describe changes in network patterns that have implications for conservation. Diversity metrics are partner diversity, vulnerability/generality, interaction diversity and interaction evenness, and distribution metrics are the specialization indices *d*′ and $H_{2}^{\prime },$ and modularity. Distribution metrics account for sampling bias and may therefore be suitable indicators to detect human-induced changes to plant–pollinator communities, thus indirectly assessing the structural and functional robustness and integrity of ecosystems. We propose an implementation pathway that outlines the stages that are required to successfully embed a network approach in biodiversity conservation. Most importantly, only if conservation action and study design are aligned by practitioners and ecologists through joint experiments, are the findings of a conservation network approach equally beneficial for advancing adaptive management and ecological network theory. We list potential obstacles to the framework, highlight the shortfall in empirical, mostly experimental, network data and discuss possible solutions.

## Abstract (precis)

Ecological networks are a useful tool to study the complexity of biotic interactions at a community level. We introduce a framework for network analysis to be harnessed to advance biodiversity conservation by using plant–pollinator networks and islands as model systems. Conservation practitioners require indicators to assess management effectiveness and validate overall conservation goals. We propose the use of several network metrics that indicate human-induced changes to plant-pollinator communities, and illustrate an implementation pathway to successfully embed a network approach in biodiversity conservation. We list potential obstacles to the framework, highlight the shortfall in experimental network data, and discuss solutions.

## Introduction

Biotic interactions characterize the biological structure of ecosystems. It is essential to monitor changes in interactions between organisms to understand the consequences of structural changes for ecosystem function and stability (McCann 2007). The complexity of biotic interactions is best studied with a network approach (Jordano 1987; Proulx *et al*. 2005), where each network illustrates a specific ecosystem function, such as pollination, seed dispersal or predation. Using a network approach allows us to depict and analyse heterogeneous, complex distribution of such functions and their roles in the ecosystem. Much progress has been made in identifying network patterns and drivers of the dynamics observed in biotic interactions. Pervasive network patterns include skewed frequency distributions of interactions resulting in a few species with many links and many species with few links (Bascompte and Jordano 2007), largely driven by species-specific abundance (Vázquez and Aizen 2003; Blüthgen *et al*. 2008), and the formation of cohesive subsets of organisms that interact more closely with each other than with other organisms in the community (modularity; Olesen *et al*. 2007; Mello *et al*. 2011; Dormann and Strauss 2014). Many studies evaluate the influence of these patterns on biodiversity and assess their contribution to the stability of the ecosystem, but the outcomes are ambiguous and, in parts, contradictory (e.g. Dunne *et al*. 2002; Vieira and Almeida-Neto 2015).

Recent advances shed light on the relationship between observed patterns and drivers of biotic interactions, which encourages the use of a network approach in disciplines outside the strict realm of network ecology. Scientists have long called for novel tools and approaches that link theory and practice (Gardner *et al*. 2009). One promising field of application of a network approach is biodiversity conservation (Memmott 2009). Indeed, several studies have used network analysis to investigate the effects of human disturbance and land use on biotic interactions at the community level, thus linking conservation and restoration biology with network ecology (e.g. Forup *et al*. 2008; Heleno *et al*. 2010; Devoto *et al*. 2012). While these insights are important for identifying the response of communities to human disturbance and ecological homogenization, they fall short of providing a strong evidence base for conservation actions. In this context, it is particularly important to determine the causal relationship between human action and temporal and spatial turnover of functional diversity in interactions. A first step was presented by Tylianakis *et al*. (2010) who discussed the use of interaction networks in conservation planning and monitoring. The authors advocated the use of certain network metrics in applied conservation and listed some caveats based on the knowledge at the time. Here, we propose to resume and go beyond this debate in light of more recent developments in network ecology and highlight strengths and weaknesses which have previously been overlooked. Our goal is to apply our theoretical understanding of biotic interactions to the field of applied conservation and restoration management.

We present a framework for network analysis to be harnessed to advance conservation action on the ground by using plant–pollinator networks on islands as a case study. Island ecosystems are perfectly suited to this purpose (Kaiser-Bunbury *et al*. 2010*b*), especially because they harbour isolated and relatively simple species communities, which can facilitate comprehensive and in-depth studies of network dynamics. In addition, island conservationists have pioneered many successful techniques and methods to mitigate threats to much of the world's threatened biodiversity, which have been subsequently applied to mainland ecosystems. Understanding and tracking the consequences of management actions on the underlying biological structure of ecosystem is key to successful conservation. Lastly, there is increasing evidence that island floras contain a large proportion of biotically pollinated species (e.g. Kato 2000; Kato and Kawakita 2004), which makes them susceptible to the disruption of their reproductive mutualisms (Traveset and Richardson 2006), despite earlier claims of high levels of self-compatibility in island plants (e.g. Barrett 1996). In the following we will (i) outline briefly the need for monitoring in adaptive conservation management; (ii) present advances in network ecology with relevance to ecosystem function and stability; (iii) suggest a thought experiment on how a network approach can guide conservation decisions; (iv) propose an implementation pathway to integrate network indicators in adaptive management and (v) synthesize this information by discussing the challenges and opportunities of using networks in applied island conservation.

## The Practitioner's Perspective: Monitoring of Conservation Management

In evidence-based conservation, practitioners rely on scientific findings to develop management strategies, direct management decisions and guide the implementation of conservation actions. Scientific methodological and ecological advances, however, are rarely used to their full potential when it comes to evaluating the effectiveness of management activities, monitoring conservation progress and adapting management approaches based on the outcome of the performance assessment (Gardner 2010).

Restoration practitioners, who manage ecological communities towards reaching ecosystem conservation objectives, require information on the ecological effectiveness of their actions (Pullin *et al*. 2013). For instance, habitat restoration in the Cape Floral Region resulted in altered feeding preferences of a generalist pollinator species leaving six specialized plant species without a pollinator (Pauw 2007). These processes are difficult to detect with conventional species-level indicators, traditional biodiversity surveys and the sole quantification of species numbers and abundances. Instead, structural, functional and biodiversity indicators are required to capture critically important yet more subtle functional changes across different spatial and temporal scales (Laliberté and Tylianakis 2010). Dependent on the specific conservation goal, practitioners require the knowledge and tools to monitor the impact of conservation measures on ecosystem services and functions; assess and validate management and financial efficacy and sustainability; reduce non-target effects and be aware of substantial changes in ecological processes as a result of human intervention (e.g. Helmus *et al*. 2014). On islands, conservation aims to control and eradicate invasive alien species, preserve current levels of biodiversity, protect ecosystem functions and their impact on human wellbeing through the provision of ecosystem services, safeguard iconic species and implement sustainable and biodiversity-friendly farming techniques. An overarching goal of all of these conservation interventions is to restore or maintain the integrity (i.e. the wholeness, resistance and resilience; Noss 2004) of island ecosystems.

To tease apart the effects of conservation on ecosystems and validate their ecological impact (see Palmer *et al*. 1997), we propose using monitoring protocols for ecological communities derived from interaction network theory. Used in combination, these tools can be mutually beneficial to conservation practitioners and network ecologists (i.e. scientists that employ interaction networks to study ecosystems, including conservation and evolutionary biologists) in achieving long-term conservation goals and generating insights into the functional response of ecosystems to human interventions. Suitable indicators aid effective monitoring and adaptive management (Noss 2004), and indicators must fulfil a list of requirements to be able to distinguish between random fluctuations and ecological signal related to ecosystem integrity (Carignan and Villard 2002). We advocate the use of structural attributes of ecological networks to be used as indicators to guide and assess conservation objectives (Kaiser-Bunbury *et al*. 2010*b*; Heleno *et al*. 2012).

## Identifying Suitable Network Indicators

**Figure 1.**
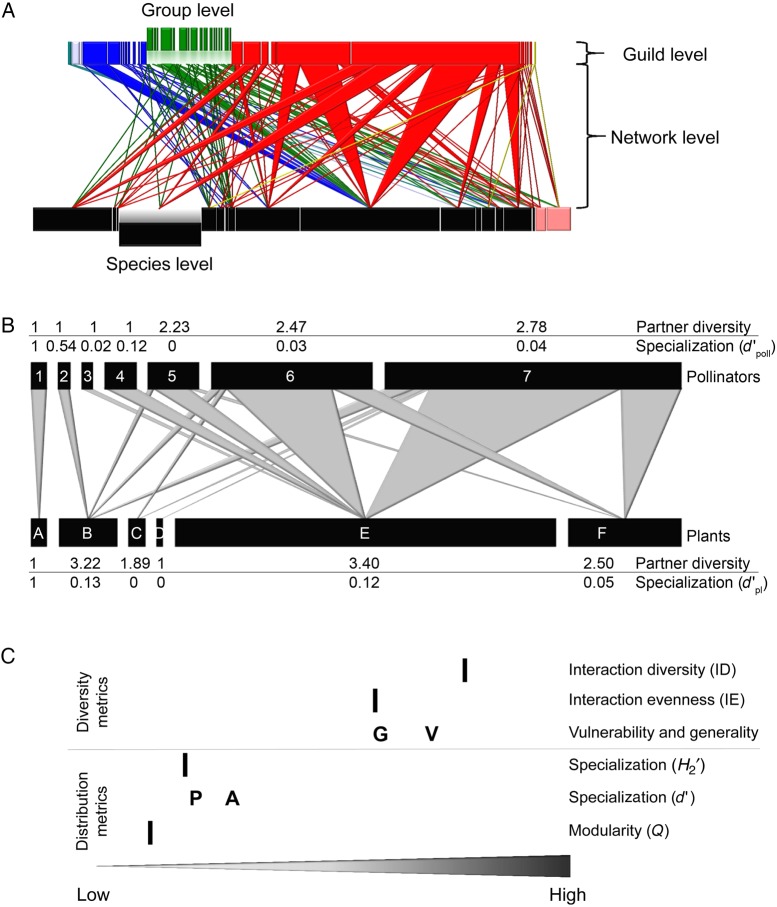
Real-world pollination web (A; data from Kaiser-Bunbury *et al*. 2012), hypothetical pollination web (B) and network metrics of the hypothetical web (C). Bipartite pollination webs (A) depict quantitative relationships between pollinators (top) and plants (bottom). Species are represented by rectangles, which are linked by wedges. The width of the rectangles reflects the relative abundance of the species, and the width of the wedges shows the relative interaction frequency between species. Pollinators are coloured by taxonomic groups (e.g. red = bees and wasps, green = flies), and plants shown in pink are exotic species, to indicate potential groupings within guilds. The real-world pollination web visualizes the hierarchical levels of the network metrics proposed in Table 1. The hypothetical web (B) illustrates conceptual differences between partner diversity of plant species (species-level generality) and specialization *d*′_poll_ and between partner diversity of pollinator species (species-level vulnerability) and specialization *d*′_pl_. Note that animal species 1–3 visit only a single plant species, thus their partner diversity is minimum (=1). On the contrary, animal species 1 is most selective (exclusive visitor of plant species *A*), and animal species 3 is least selective in terms of the distribution of all pollinators, hence *d*′ declines accordingly from species 1 to 3. For animal species with a single individual, partner diversity always equals one, whereas *d*′ can vary between zero and one depending on the exclusiveness of the selected plant species. Other network metrics (C) of the hypothetical web describe the diversity and the distribution of interactions. With higher generalization, the generality (G)/vulnerability (V) increases whereas complementary specialization *d*′ decreases (P, plants; A, animals).

To employ a network approach in biodiversity conservation, we need to understand the relationship between network properties and ecological processes, e.g. during succession. Interaction networks are schematics of interacting species in a community (Fig. 1A), and their properties describe the organization of such interactions at the community level (Vázquez *et al*. 2009*a*). By identifying and quantifying general patterns of organization in interaction networks, network ecologists describe the structure of networks and draw conclusions on ecological and evolutionary processes (Bascompte and Jordano 2007). Much progress has been made to determine the structure of networks by describing aggregate network statistics (Jordano 1987; Bersier *et al*. 2002; Tylianakis *et al*. 2007; Blüthgen *et al*. 2008) or by exploring the role of ecological variables in shaping changes in interactions over space and time, i.e. network dynamics (Herrera 1988; Petanidou *et al*. 2008; Junker *et al*. 2010, 2013; Benadi *et al*. 2012; Kaiser-Bunbury *et al*. 2014). The search for universal drivers of network patterns has generated a plethora of increasingly refined studies identifying abundance and species composition (Kunin 1997; Stang *et al*. 2006; Vázquez *et al*. 2009*b*; Kaiser-Bunbury *et al*. 2014), trait matching (Stang *et al*. 2006, 2009; Eklöf *et al*. 2013) and temporal and spatial co-occurrence of interaction partners (Olesen *et al*. 2008; Dupont *et al*. 2009; Kaiser-Bunbury *et al*. 2009, 2010*a*; Vázquez *et al*. 2009*b*) as the main determinants underlying network structure. Additional insight comes from applied studies that investigated the influence of invasive alien species (Aizen *et al*. 2008; Valdovinos *et al*. 2009; Junker *et al*. 2011; Kaiser-Bunbury *et al*. 2011; Santos *et al*. 2012; Traveset *et al*. 2013; Albrecht *et al*. 2014; Stouffer *et al*. 2014) and habitat restoration on plant–animal interaction networks (Forup *et al*. 2008; Henson *et al*. 2009; Heleno *et al*. 2010; Devoto *et al*. 2012), and those that took into account general ecological principles, such as niche theory and interspecific competition, when modelling species extinction scenarios (Benadi *et al*. 2012, 2014; James *et al*. 2012; Bewick *et al*. 2013).

**Table 1.** Network metrics suitable as indicators for conservation effectiveness. *Source*: Bersier *et al.* (2002), Blüthgen *et al*. (2006) (*H*_2_′) *d*′); Blüthgen *et al.* (2008), Dormann *et al*. (2009); Blüthgen (2010); Dormann and Strauss (2014) (*Q*); Olesen *et al*. 2007 (modularity).

Hierarchical level	Metrics	Metric description	Implications for conservation
(a) The following four metrics describe the diversity of interactions at three levels (network, species, guild). Higher diversity generally suggests higher richness and evenness of species and/or higher generalization of species. Higher diversity is assumed to increase the robustness against species losses or temporary fluctuations. While these indices seem straightforward in their interpretation, they strongly depend on sampling, species abundances and the completeness of information, confounding direct comparisons across species and networks.			
Network level	Interaction diversity (ID)	Weighted ID across a network, best calculated as the exponent of the Shannon Entropy *H*_2_ across all links. Since links are weighted, ID is the quantitative analogue to the total number of links.	Higher ID implies higher community stability. However, if alien species account for a large proportion of the ID, resource competition between native and alien species may be high, potentially compromising the stability of native communities or quality of the ecosystem function.
	Interaction evenness (IE)	Homogeneity of interaction frequencies across all links in the network, with high values reflecting more uniform spread of interaction among the species in the community. Its qualitative analogue is connectance.	If some species and their links dominate the communities, while most others are rare, IE may be low. This may be a consequence of invasion processes or habitat degradation. On the other hand, evenness may increase when many rare species become locally extinct (i.e. homogenization), coinciding with poor ID.
Species level	Partner diversity	Diversity of interaction partners for each species. It is the quantitative analogue to the qualitative species degree, i.e. the richness of interaction partners.	Individual species—similar to communities—may benefit from interacting with a diverse set of resources or mutualistic partners. High partner diversity would reduce the reliance on a few, specialized species, thereby increasing the robustness of species to stochastic or anthropogenic disturbance. Low levels of partner diversity and, thus, generality or vulnerability may indicate risks by human-mediated disturbance that require conservation action to counteract loss of functional quality.
Guild level	Vulnerability and generality	The mean diversity of interaction partners across all species within a guild (plants or animals). Hence, a summary of plant and pollinator species partner diversity, respectively.	
(b) The following three metrics characterize the distribution of interactions relative to each other, namely their mutual exclusiveness. The metrics increase in value when species (or sets of species) are highly specialized on specific partners (high partitioning). Unlike the metrics above (a), this concept is independent of the completeness of information and number of observations per species, and can be compared directly across different species and networks.			
Network level	Specialization $\left({H_{2}}^{\prime }\right)$	Link complementarity across all species. High specialization indicates high dependency of each species on a few exclusive partners. Low specialization indicates higher functional redundancy.	All diversity-related metrics in (a) increase with the number of observations per species and sampling intensity. ${H_{2}}^{\prime }$ and *d*′ quantify the degree of specialization independent of species frequency and sampling. ${H_{2}}^{\prime }$ and *d*′ are therefore ideally suited to directly compare different networks $\left({H_{2}}^{\prime }\right)$ or species within a network (*d*′), e.g. specialization levels of alien versus native sets of species, or heavily modified with natural networks.
Species level	Specialization (*d*′)	The exclusiveness of a species’ partner spectrum compared with other species in the network. This metric can be altered to express a comparison of realized interactions with the availability of partners or resources. Species with low species-level specialization indicate opportunistic partner selection compared with other species in the network.	*d*′ can be used to assess the level of exclusiveness of a species in terms of its interaction partners (i.e. its specialization as a non-overlap with the other species in the same guild), with implications for competition for resources (pollinators) or services (plants) between species in the network. Invasive alien species with high species-level specialization may be a lower threat, or may even benefit a few native species that have lost specialized pollinators, than those with a very broad partner spectrum, imposing competitive pressure on a range of species. Network-level specialization $\left({H_{2}}^{\prime }\right)$ summarizes all species *d*′, which are weighted by their overall abundances.
Group level	Modularity (*Q*)	Modules are aggregates of interacting species. Modules help to visualize groups of species that share interactions more frequently within modules than across modules.	Modularity helps to distinguish between topological roles of species in networks, such as species that are responsible for within- and between-group cohesion, and peripheral and central species key to the structural integrity of networks. Information on the origin and ecology of these species can guide management decisions. By strengthening certain connector species modularity could be reduced at the network level while increasing connectivity between hubs. Further, modules can be used to locate the lack of functional redundancy and complementary. Modularity is partly related to specialization metrics above.

Based on this empirical and theoretical background, Tylianakis *et al*. (2010) proposed several network properties that could be used as indicators of the status of pollination networks. The implications for ecosystem integrity of some of these metrics and patterns are, however, controversial. Asymmetries are assigned a stabilizing role (Bastolla *et al*. 2009; Rohr *et al*. 2014), but other studies argued against such mechanisms (Benadi *et al*. 2012; James *et al*. 2012). Similarly, connectance was proposed to stabilize and destabilize an ecological community (Dunne *et al*. 2002; James *et al*. 2012; Sentis *et al*. 2014; Vieira and Almeida-Neto 2015). These and other metrics commonly used to describe network patterns, such as species degree (i.e. number of links per species) and nestedness, should be considered with care. They tend to reflect primarily variations in the incompleteness of the information about each species' links with potential partners (sampling limitation) rather than their specialization *per se* (Vázquez *et al*. 2009*a*; Chacoff *et al*. 2012). For example, nestedness is a pattern that largely reflects asymmetries in abundances (Blüthgen *et al*. 2008; Blüthgen 2010). Rarity is a clear and important concept highly relevant for conservation; it should therefore be evaluated explicitly and independently of its confounding effects on a network pattern (Blüthgen *et al*. 2008; Dorado *et al*. 2011). Whether, for instance, a log-normal abundance distribution, which generates a nested pattern, is *per se* more or less stabilizing than a uniform distribution remains unresolved, thus limiting the applicability of such concepts. Network ecologists therefore employ a null model approach to tease apart changes in species abundance (and sampling effects), species diversity and changes in species generalization (Vázquez and Aizen 2003; Blüthgen *et al*. 2008). Quantitative null models (e.g. Patefield's algorithm) control for sampling bias, abundance and diversity, and are thus important for the unbiased interpretation of changes in network metrics.

Here we introduce several network metrics on different hierarchical levels, which we believe are the most suitable indicators for conservation effectiveness because of the ecological characteristics of the indicators, sound empirical and theoretical support, conceptual similarities to well-established diversity indicators and computational ease (Table 1). The proposed set of network metrics is not exhaustive and should be revised in light of future advances in the understanding of ecological processes underlying network structure; other metrics, such as betweenness and closeness from the concept of centrality (e.g. Martín González *et al*. 2010; Gómez and Perfectti 2012; Mello *et al*. 2015) and metrics that describe structural robustness to secondary extinctions (Memmott *et al*. 2004; Kaiser-Bunbury *et al*. 2010*a*), or detailed investigations on the microstructure of networks and the importance of individual links (Vázquez *et al*. 2009*b*; Junker *et al*. 2010; Kaiser-Bunbury *et al*. 2014), may also be suitable to address the many biological questions and conservation challenges. More empirical and theoretical studies are needed to explore these concepts in more detail. We decided therefore to propose only a small, exemplary set of quantitative network metrics, which we believe could be most effective in facilitating decision-making in conservation. We selected network indicators based on two general criteria: first, weighted metrics are preferable to unweighted metrics (Bersier *et al*. 2002; Banašek-Richter *et al*. 2004). Weighted metrics account for the quantitative importance of different species and their interaction partners, whereas unweighted metrics assume that every species is the same, irrespective of the frequency with which it interacts in the network. This holds true if suitable measures of interaction frequency, interaction rates or other measures of interaction strength, which correspond to functional relevance, are used to calculate weighted network metrics (Vázquez *et al*. 2005; Sahli and Conner 2007). Second, relatively sensitive and variable metrics are required to detect temporal and spatial variations (Gardner 2010), which support the previous argument that metrics such as connectance and (unweighted) nestedness are unsuitable as indicators of network patterns because the metrics' underlying drivers—unequal relative abundances—are virtually ubiquitous and remain therefore relatively constant (Heleno *et al*. 2012, but see Tylianakis *et al*. 2010).

We distinguish between two main network attributes, analogous to the frequently-used concepts of α- and β-diversity: ‘diversity’ and relative ‘distribution’ of interactions (Fig. 1A, Table 1). Interaction diversity metrics at the network, species and guild levels correspond to elements of α-diversity, such as species richness and evenness within and across hierarchical levels. Composition and grouping of interactions within and across hierarchical levels are expressed by specialization at the network and species levels, and the aggregation of groups of closely interacting species (modules, Fig. 1A). To improve the ease of applicability, we describe their ecological interpretation and advocate the use of appropriate analyses (i.e. null models and/or rarefaction) to disentangle different drivers of the pattern and control for sampling effects.

## Network Metrics Aid Conservation Decisions: A Thought Experiment

For a fictive plant–pollinator network on an island, assuming that it comprises native (i.e. desirable) species only, one general conservation objective may be to preserve the diversity of links to increase functional robustness—defined here as the ability of plant–pollinator communities to respond to changes while maintaining relatively normal functional properties, i.e. pollination quality and quantity, and feeding behaviour by pollinators—against possible disturbances. This objective is best validated by the ‘interaction diversity’ (ID) metric on the network level and ‘partner diversity’ on the species level (Table 1). Like species diversity, ID has two components: number of links (richness) and homogeneity of relative interaction frequencies across the links (interaction evenness, IE). A more even distribution of links is likely to be associated with higher functional robustness, given that risks of losing a link or entire species, or fluctuations of frequencies, are spread evenly across the network. This argument gains theoretical and empirical support by classical concepts on the relationship between species diversity and stability (insurance hypothesis: Yachi and Loreau 1999; portfolio effect: Thibaut and Connolly 2013). Additionally, functional and structural robustness increase with the heterogeneity of interaction partners and their complementarity in terms of environmental responses (response diversity; Elmqvist *et al*. 2003; Hooper *et al*. 2005). Diversity not only increases stability but also the functional performance level (Hector *et al*. 1999). In pollination, similar relationships were shown in agricultural systems, where pollination success increased with higher functional complementarity of pollinator species (Klein *et al*. 2003; Hoehn *et al*. 2008; Blüthgen and Klein 2011).

The conservation objective of maximizing ID may be accomplished via three management trajectories that target (i) increased species diversity, (ii) increased abundance (density) of each species and, thus, higher likelihood for species to interact and/or (iii) lower specialization of the existing species. The former two trajectories may be pursued by monitoring classical biodiversity metrics and employing rarefaction analysis to control for methodological bias. Simple network metrics and visualization tools may be used depending on the data collection method, but specific network analysis is not required. To identify specialization effects in interactions, however, a suitable network metric should be independent of variation in total species diversity and abundance. The metric that satisfies this requirement is the complementary specialization $H_{2}^{\prime },$ which quantifies the specialization–generalization continuum of the community for fixed diversity and abundance based on the selectivity of species (Table 1). The continuum from specialization to generalization can be defined in several ways, for example as diversity of association partners (niche breadth)—ranging from few to many partner species—or as selectivity (niche complementarity)—from highly selective to highly opportunistic choices of available partners (Fig. 1B and C). Both concepts are inversely related, that is high selectivity of a species decreases the diversity of its partners. In interaction datasets that are limited by sampling, however, partner diversity simply increases with the number of observations, whereas selectivity (e.g. $H_{2}^{\prime },$ *d*′) can be determined independently of the completeness of interactions observed. Both high specialization and high selectivity suggest a high dependency on certain partner species and, thus, vulnerability to their losses (Fig. 1B and C). Note that higher generalization (low $H_{2}^{\prime }$) may increase functional robustness, but it may not be desirable *per se* and comes at a cost: generalists often show lower performance levels than specialists at particular activities, such as pollination (e.g. Larsson 2005). At the species level, the specialization metric *d*′ can be used to identify species that are more specialized and possibly vulnerable to extinction, or those that are more generalized (Fig. 1B, Table 1). Together with partner diversity, these species-level metrics may improve our understanding on the roles of individual species in networks.

These metrics may be particularly relevant in a conservation context on islands where all-native systems are the exception and species attributes, e.g. origin or conservation status, are important factors directing management decisions (Noss 2004). In contrast to the native community example above, conservation objectives for communities with alien or invasive species in a network generally aim to reduce total abundance, partner diversity, ID and IE. Species origin is also important for the interpretation of network metrics with regards to functional robustness and ecosystem integrity. For instance, as interactions between a few endemic species gain in strength, IE declines. This may be considered beneficial for maintaining co-evolved ecosystem dynamics and functional diversity despite a perceived decline in structural robustness due to reduced IE. In turn, increased IE can be a consequence of alien species that drive homogenization of a community, most commonly an undesirable effect from a conservation perspective. In such a scenario, a measure of functional performance (e.g. fruit or seed set) or robustness (competition or facilitation between native and alien species) could be used to verify the desired conservation effect.

Having identified network indicators and presented the underlying rationale of linking interaction network patterns to ecosystem functioning and integrity, the next step involves the successful incorporation of network methods and metrics in conservation. This phase builds on the engagement of network ecologists in conservation management and practitioners who embrace novel scientific ideas and methods (Kaiser-Bunbury *et al*. 2010*b*, 2015). We propose an implementation pathway that uses best scientific and applied approaches in both disciplines and advocates mutually beneficial collaborations.

## A Framework for Using Networks in Conservation

**Figure 2.**
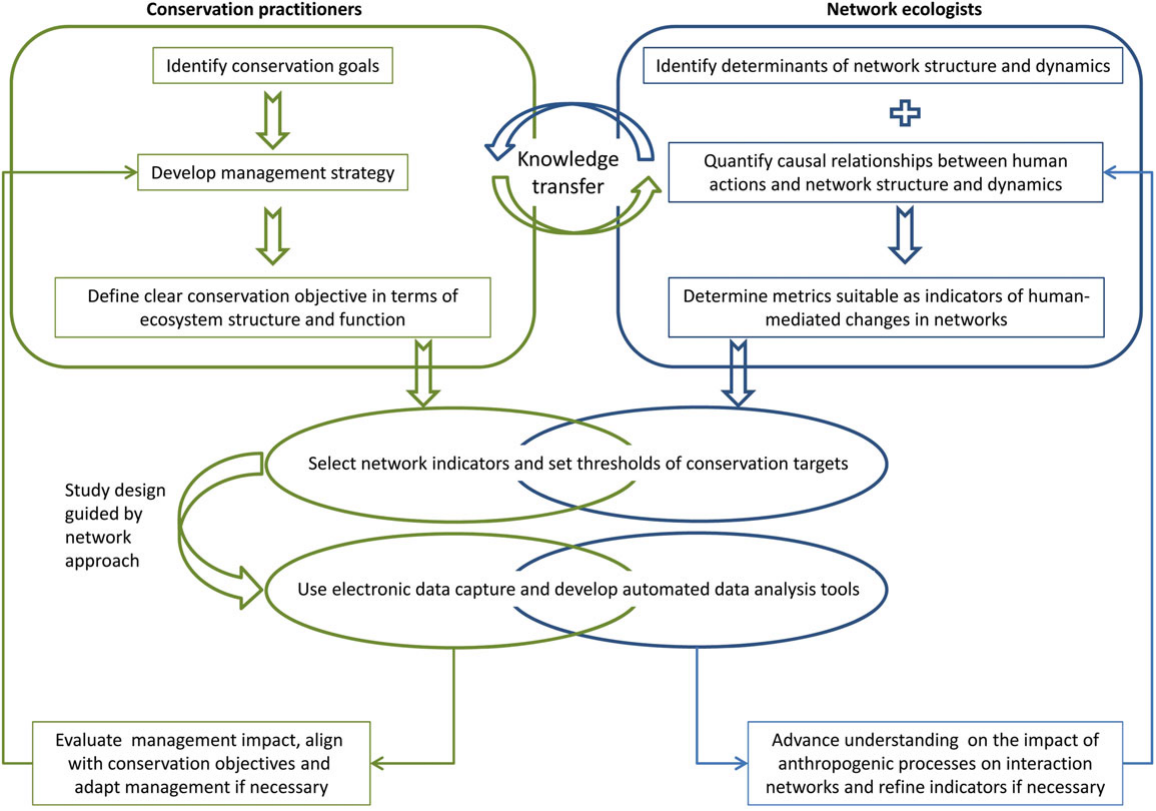
Pathway to implement an interaction network approach in biodiversity conservation. Practitioners undergo a multi-stage process to define conservation objectives specific to one ecosystem function. Concurrently, network ecologists determine the causal relationship between human action and network patterns, and identify suitable metrics as indicators to assess conservation management effectiveness. Selecting indicators, setting thresholds and choosing the appropriate methodology for data collection are jointly carried out between ecologists and practitioners to ensure rigorous experimental setup. Findings are used for adaptive management by practitioners and for refining network analysis by ecologists.

To integrate network indicators in adaptive management, the ecological message derived from the interpretation of the indicators must be aligned with conservation goals (Fig. 2). Prior to engaging with network ecologists, conservation practitioners identify conservation goals, develop a management strategy and define clear conservation objectives and outcomes (see also Noss 2004; Gardner 2010). This is not a trivial process; objectives have to clearly identify the functional role (i.e. niche) that should be altered through intervention as networks can help to unravel the species' roles in one functional niche dimension at a time and the effects on other functional groups may not be beneficial for the functional niche under observation (Pocock *et al*. 2012). Additional life-history parameters, species traits such as the responses to the environment or their conservation status may be orthogonal to the functions displayed in the network, thus requiring a different methodological and/or analytical approach. To prioritize conservation action in the face of uncertainty it may be best to use tools that analyse the multivariate functional space based on decision theory (see McCarthy and Possingham 2007). Clear conservation objectives will then provide the basis for selecting network indicators and setting threshold values of conservation targets.

In parallel, community experiments and theoretical approaches further understanding on causal relationships between network metrics and targeted conservation actions, e.g. habitat restoration, invasive species control and protected area management (Fig. 2). The scientific advances derived from the experiments may be incorporated into indicator selection and threshold definitions to enable practitioners to evaluate how best to achieve continued progress towards long-term conservation goals (validation monitoring; Gardner 2010). We believe that it is central to the success of the process that this stage is implemented through shared first-hand experience between practitioners and ecologists. Only if conservation action and study design are aligned can network indicators be appropriately employed for the validation monitoring. To facilitate data collection and prompt analysis, available software can be used for electronic data collection and developed further for real-time analysis. For example, mobile data capture and visualization software (e.g. as a pollinator field identification tool) such as CyberTracker™ (www.cybertracker.org) can be employed on hand-held, rugged field computers, allowing for rapid data collection and consequent analysis with suitable libraries (e.g. bipartite; Dormann *et al*. 2008) in the statistical software package R (R Core Team 2014). The outcomes are mutually beneficial for practitioners and ecologists (Memmott 2009): the conservation effectiveness can be monitored and evaluated, allowing for adaptive management based on ecosystem functions. Ecologists benefit from large-scale ‘experiments’ and datasets that help to refine network metrics and predictions on anthropogenic impact on network patterns (e.g. Devoto *et al*. 2012).

## Challenges and Opportunities of Interdisciplinarity in an Island Setting

Studies of island ecosystems have been instrumental to our understanding of fundamental processes in ecology and evolution (Warren *et al*. 2015). Equally, endemic island biotas are in the centre of the Holocene extinction (Whittaker and Fernández-Palacios 2007). While ecologists and evolutionary biologists rush to study what is left of natural processes in island ecosystems, conservation practitioners pioneer management techniques and contribute significantly to the field of conservation biology by trying to conserve biodiversity hotspots that are exposed to severe environmental threats. Islands therefore provide a perfect interdisciplinary interface for scientists and practitioners, an experimental set up of discrete, manageable and replicated biological communities (Warren *et al*. 2015).

While the island setting is ideally suited to overcome any impediments in adjusting the two approaches, the utility of a network approach in conservation needs to be assessed in light of the limitations of the two disciplines. One of the most pervasive limitation is the fact that network data are an incomplete reflection of the links that are potentially relevant in an ecosystem, with rare species having higher information deficits than common, ubiquitous ones (Vázquez *et al*. 2009*a*). This may overestimate the rare species' specialization and dependency on specific partners, and underestimate their potential role in the network. Rare species are a classical conservation target, and it is important to better understand their potential functions, environmental niche and interaction partners. Research should use a more targeted approach to better understand the functional role of rare species in communities. One way of overcoming the bias of community studies may be by incorporating external data (e.g. specific food plants, life-history traits, information on phenophases) on rare target species specifically collected for species-based management, or derived from theory (Petanidou *et al*. 2008; Kaiser-Bunbury *et al*. 2010*a*; Dorado *et al*. 2011). For example, pollen loads carried by pollinators indicate whether rarely sighted flower visitors are in fact rare and highly specialized pollinators (Bosch *et al*. 2009). A similar method is applied in the compilation of classical food webs where links are typically ‘inferred’ (from literature or expert knowledge), not observed. This method could be useful to supplement gaps in empirical networks with theoretical knowledge, albeit the potential for considerable methodological pitfalls. To date, limited information on rare species in plant–pollinator networks continues to be prevalent, compromising the validity of ecological conclusions derived from under-sampled networks.

The rare-species dilemma is closely linked to two other inherent limitations of a network approach: recording interactions comes at a relatively high cost and with a number of methodological pitfalls (Tylianakis *et al*. 2010; Devoto *et al*. 2012). Collecting data on species interactions at the community level is also time and labour consuming. It could be argued that the conservation of networks is warranted despite the higher costs and time investment, given the aforementioned benefits of monitoring and analysing interactions, in addition to species diversity. Conservation actions, however, are disproportionately underfunded, and even if conservation would suddenly experience a sharp rise in funding, interaction data may not make the priority list given the apparent cost-inefficiency. Similarly, there is no one-fits-all method that can be applied to collect data on most types of interaction and habitats. In short, network data collection must be cost and time effective and methodologically simple, clear and ideally widely applicable to be of any use to practitioners. Hegland *et al*. (2010) suggested rarefying data collection without compromising data resolution to reduce costs, but this may accentuate the rare-species problem as those are not detected in under-sampled networks. Alternatively, network theory and models may be able to assist by determining the underlying drivers of pairwise interactions (Kaiser-Bunbury *et al*. 2014). Once identified, practitioners could collect data primarily on the drivers instead of interactions, and these data may be used as a proxy to deduce network patterns and processes. While this scenario may be unrealistic based on the current level of knowledge, we are confident that advances in understanding interaction dynamics will strengthen our ability to predict network patterns and processes from theory in the near future.

Another caveat of using plant–pollinator networks is that, as in most community studies, data on the frequency of interactions are assumed to mirror the quality and quantity of pollination (Vázquez *et al*. 2005). In weighted approaches in general, including diversity indices and quantitative network analyses, rare species play a minor role by definition, implying that low abundance is associated with low relative functional importance. This assumption, however, might not always withstand rigorous testing (Genini *et al*. 2010; King *et al*. 2013), suggesting that detailed empirical studies on the ecological role of species are necessary to determine the long-term outcome of conservation trajectories.

While we may not be able to meet this target with our current knowledge, the presented framework (Fig. 2) outlines a possible trajectory towards effectively using network approaches in conservation action. Additional information on network assembly and disassembly, fluctuation of interactions across species and mutual dependencies would further strengthen our ability to predict networks and use network metrics in conservation. To overcome the ambiguity in interpreting some network metrics, causal relationships between changes in network patterns and functional robustness remain to be tested experimentally and data on long-term dynamics are needed. The fact that island faunas and floras are generally less diverse than mainland communities at the same latitude and altitude (Schleuning *et al*. 2012), resulting in interaction networks of lower complexity, especially in the tropics, further facilitates the prediction of network patterns (Kaiser-Bunbury *et al*. 2014). This does not, however, exclude the transfer of the proposed network approach to conservation practices that address ecosystem degradation in mainland areas. Networks have been successfully employed to assess restoration of ancient heathlands (Forup *et al*. 2008), meadows (Albrecht *et al*. 2007) and ancient pine forest in the UK (Devoto *et al*. 2012). Similarly, network concepts have been developed and tested in the mainland conservation context of fragmented landscapes (e.g. Hagen *et al*. 2012), recovery of agricultural land (e.g. Russo *et al*. 2013) and landscape management for conservation (e.g. Ferreira *et al*. 2013).

A shift in conservation priorities from species-based approaches to ecosystem-based approaches has been widely advocated (e.g. Millennium Ecosystem Assessment 2005). While network properties are inherently ecosystem-based indicators, they can equally be used to assist in traditional conservation approaches prioritizing rare, threatened, keystone or charismatic species by contextualizing their roles and identities in the ecosystem. Networks can not only illustrate the target species' functional role in the system, but also their relevant resources, competitors and potential enemies—note, however, that identifying functional roles of species may require data from several seasons, even in a depauperate island system, given the large inter-annual turnover of the pollinator community (e.g. Petanidou *et al*. 2008). For example, particularly problematic invasive species may be identified as those that are associated with a broad spectrum of endemic species, or which have a disproportionally high frequency of interaction with endemic species, thereby outcompeting native interactions. Incorporating these findings into conservation may mean questioning current management decisions and potentially adjusting the general conservation rationale. By identifying the functional role of alien invasive species in degraded ecosystems, conservationists have sparked a debate on the role of novel ecosystems in conservation (Hobbs *et al*. 2009; Kueffer and Kaiser-Bunbury 2014). For example, many natural and agricultural plant communities lack native pollinators due to land-use change and overuse of chemicals in agro-ecosystems, and introduced honeybees ensure pollination services, albeit of lower quality (Garibaldi *et al*. 2011).

A relevant decision-making trade-off in conservation addresses the conflict between management towards greater stability of functions by protecting and promoting the performance of generalists, and management that prioritizes specialists as superior performers for certain functions despite the greater risk of extinction (see also Devoto *et al*. 2012). Often these decisions are made indirectly by funding schemes which focus either on species-list conservation or an ecosystem stability approach (Possingham *et al*. 2002; Pullin *et al*. 2013). Similar questions arise if conservationists are confronted with the decision on whether to conserve unique links, exclusive links or parts of greater modules. It is beyond the scope of this work to present answers to these questions; however, it is important to continue the debate and present solutions throughout the process of assimilation between the two disciplines. Once the interaction network approach has been adopted by practitioners, there is the potential to expand the concept beyond species–species networks, for example, to include habitat networks on a landscape scale (Baguette *et al*. 2013) or employ network and decision theory to optimize conservation strategies (e.g. Caplat *et al*. 2012).

We have illustrated, from our experience in island systems, a promising pathway to apply knowledge on plant–pollinator interaction networks to aid conservation actions and improve management effectiveness. Despite a shortfall in (experimental) field data to determine the response of networks to ecological restoration and/or degradation, the main challenge of the relationship between network ecology and conservation lies at the interface between the two disciplines. We believe that ecological theory and network tools are sufficiently advanced to determine and measure the desired ecological state of many conservation aims. Equally, many conservation practitioners excel in harnessing traditional monitoring tools in adaptive management to assess effectiveness and validate the impact of their actions. There is, however, limited cross-disciplinary exchange between network ecologists and practitioners, to the detriment of the advances of both disciplines. Island ecosystems present the perfect ‘laboratories’ to merge these disciplines and test new theoretical and applied concepts to address the challenges of the Anthropocene in terms of nature conservation and advancing our understanding of highly dynamic and complex ecosystem processes.

## Sources of Funding

C.N.K.-B. was funded by the German Research Foundation (DFG; KA 3349/2-1).

## Contributions by the Authors

C.N.K.-B. conceived the ideas, designed the implementation pathway and wrote a first draft. Both authors developed the conceptual classification of network metrics as indicators and contributed to writing.

## Conflict of Interest Statement

None declared.
